# Examination of Summer Campers’ Physical Activity Interest and Behavior

**DOI:** 10.3390/ejihpe10010018

**Published:** 2019-11-20

**Authors:** Joseph Otundo, Bennie Prince

**Affiliations:** College of Education Health Professions, School of Counseling Human Performance Rehabilitation, University of Arkansas Little Rock, 2801 S University Ave, Little Rock, AR 72204, USA; jootundo@ualr.edu

**Keywords:** health, physical activity, prosocial patterns, antisocial patterns, children summer camps, psychological theory, social development theory

## Abstract

This study has provided insight into the complex relationship between situational interest, the social environment, competence, and behavior. This quasi-experiential design included a pre-test questionnaire, intervention activity of throwing and catching a football, and post-test questionnaire administered to forty children aged 7–13 enrolled in a summer camp. The results of this study supported a theoretical approach that hypothesized that competence positively influences situational interest. The implications for summer camp owners and counselors support providing an environment that is supportive of elementary-age children participating in an established curriculum that results in positive interactions and activities where summer campers can perceive that they are competent.

## 1. Introduction 

Every year, over 10 million children of all ages attend summer camps to learn and engage in sports and other activities that help develop social skills, communication skills, problem-solving skills, and life skills [1,2]. A summer camp is a youth development organization, supervised by professional adults, that strives to foster personal growth for children by providing them fun, safe educational/recreational programs, outdoor experiences, and group activities while away from home during the summer months [1,2].

Researchers [3] support the argument that summer camps have a positive influence on peer interactions, the development of personal skills, and physical growth. However, it has also been reported that some campers are not only disinterested in camping activities, but also exhibit anti-social behaviors [2,4]. Prosocial behavior is an issue that ought to be emphasized among children attending the summer camps. Parents are often concerned about the safety and socialization that occurs at the summer camps. Having said this, it is important that activities taught at summer camps be designed based on a curriculum that encourages prosocial skills in addition to motivation based on personal and situational interest.

Camp counselors and directors are entrusted with the responsibility of providing a safe and conducive environment for all campers, even though it is often challenging to predict the anticipated behavior of each camper. In a study of children whose mothers were incarcerated, researchers [5] found higher levels of antisocial behavior among the children who did not have social support. Camp organizers are also concerned with the identification and implementation of activities that motivate campers, as well as eliminating any form of anti-social behavior. Researchers have often cited a lack of interest as a major hindrance to motivation [6]. In a study with children [6], the results point to an association between interest and anti-social behavior. This study will examine the relationship between interest theory, basic psychological theory, and social development theory. This investigation will explore the interaction between summer campers’ situational interest, personal interest, and competence impact on their physical activity.

### 1.1. Interest Theory

Interest is defined as a relatively continuing tendency to re-engage with particular content that may include objects, events, and ideas [7]. It is well-documented that students’ motivation is associated with the level of interest, as well the social support they get from the learning environment [8]. According to both 2002 and 2013 studies on students’ interest in physical activity, this factor correlates highly with the experiences, knowledge, exposures, and situational factors in the learning environment [7,8].

Researchers have hypothesized two types of interest, that is, personal and situational interest [7,8,9]. Personal interest is a relatively enduring predisposition to attend to objects, events, and ideas [9]. In addition, it develops over time and is associated with positive feelings, increased value, and knowledge [10]. Individuals are interested in tasks for which they are knowledgeable and competent. The individual then places value on these tasks and is personally interested in these tasks. In addition, situational interest is environmentally influenced, involves an affective reaction, and focuses on the individual’s reaction [6,8]. The learning environment, feelings, and individual reactions to the situation influence situational interest. Research points to five indicators of situational interest: novelty, attention demand, instant enjoyment, exploration intention, and optimal challenge [8]. Besides these five indicators, the study suggests that social factors, specifically basic psychological needs, help to explain the social aspects of interest theory.

### 1.2. Basic Psychological Theory

A study on basic psychological theory [10] posits that an individual’s motivation and situational interest are influenced by the learning and sports environment that uphold the three basic psychological needs of autonomy, competence, and relatedness. Autonomy is experienced when students feel a sense of volition about the choices and decisions they make in a specific context [11]. An autonomy-supportive environment tends to nurture inner motivational feelings, rely on controlling approaches to teaching, and acknowledge an individual’s perspectives and feelings [12]. Current research on self-determination theory supports the focus on satisfaction of basic psychological needs or on studying the motivational processes [13]. Self-determination theory (SDT) of human motivation, personality, and well-being is applicable across many life domains. A critical component of SDT is that all human beings have three basic psychological needs that are critical for autonomous motivation, wellness, and learning [13].

Relatedness is having strong interpersonal interactions and trusting relationships. Therefore, in a relatedness supportive environment, campers are motivated when they interact amongst themselves (including forming sports teams), and with the camp counselors. Lastly, competence is the need to be effective in one’s pursuit and interactions with the environment [11]. Similarly, it is the inherent desire to exercise one’s capacities to master the environmental challenges. Competence may be acquired after learning various fundamental skills in order to improve one’s ability to perform well. The process of learning to be competent or perform well might lack motivation from the learner to continue [14].

Even though the social environment impacts situational interest, it is not clear how children acquire specific behavior traits and how these traits are associated with interest. To explore this aspect, we utilized social development theory.

### 1.3. Social Development Theory

Proponents of social development theory maintain that children’s patterns of behavior are influenced by socializing units [15,16]. Patterns of behavior (prosocial and antisocial) are developed from socializing units such as family, school, religious organizations, other community institutions, and peers [15,16]. Besides the school environment, summer camps are avenues that afford children the opportunity to interact, develop social skills, and engage in physical activities [17,18]. In these two studies, it is suggested that in children socialization, perceived opportunities for involvement in physical activities reinforce what they get from participating in these interactions.

When socializing processes are consistent, a social bond develops between the individual and the social unit. Once established, the social bond has the power to affect behavior, independent of the constructs. Bonding involves attachment to the social unit and therefore aspiring to do what conforms to the norms of the social unit [17,18]. When bonding, individuals do not care whether their behavior is acceptable or unacceptable in the wider society. The behavior of an individual is therefore prosocial or antisocial, depending on predominant behaviors, norms, and values.

Attending summer camps has numerous benefits for the attendees. However, there seems to be a problem within their motivation and social behavior in attending the summer camp and participating in activities provided. Research investigating the relationship between interest, social factor, and behavior patterns among summer campers is scarce.

Therefore, the purpose of this study was to investigate the relationship between personal interest, situational interest, and prosocial and antisocial behavior. The following hypotheses were investigated:

H_1_: There is an association between personal interest and situational interest;

H_2_: There is a relationship among personal interest, situational interest, and competence;

H_3_: Prosocial behavior is a mediator between personal interest and situational interest.

This was a cross-sectional study conducted with children (N = 40) enrolled in a summer camp. Personal Interest Surveys [19] were administered to campers at the beginning of a football activity at a summer camp. Campers then participated in the intervention activity of throwing and receiving in football. The Situational Interest Survey [20] and Child Behavior [21] surveys were administered at the end of the activity.

## 2. Method

### 2.1. Participants

The participants were 40 elementary children aged 7 to 13 years old (M = 9.13, SD = 1.60). The participants were enrolled in a three-week summer camp at a four-year university in the southeastern part of the USA. The sample population consisted of 57.5% boys and 42.5% girls. In total, 65% of the participants were Caucasian, 20% were African Americans, 10% were Hispanic, and 5% were others.

### 2.2. Instruments

We used the following three scales to collect data: the Personal Interest Scale [20], the Situational Interest Survey [20], and the Child Behavior Scale [21]. The Personal Interest Scale [19] measured the situational interest in secondary school math; however, embedded in this instrument are questions relating to specific techniques an instructor might use to engage students in activities. The Personal Interest Scale [19] was modified to suit camping experience. An example item from the Personal Interest Survey [19] is “I am excited about doing this activity.” The six items of the Personal Interest Survey [19] were measured on a 5-Likert scale (1-strongly disagree to 5-strongly agree).

The Situational Interest Survey [20] was used to measure the levels of students’ feelings about each item in terms of the activity they are experiencing. The 24 items of the Situational Interest Survey [20] were measured on a 5-Likert scale (1-strongly disagree to 5-strongly agree). An example item from the Situational Interest Survey [20] is “This activity is interesting.”

The Child Behavior Scale [22] measures children’s aggressive, withdrawn, and prosocial behaviors. The Child Behavior Scale [21] was measured on a 5-Likert scale (1-never to 5-very often). An example item from the Child Behavior Scale [21] is “How often do you yell at other kids during activities in the summer program?” 

Cronbach’s alpha for personal interest and situational interest were highly reliable at α = 0.936 and α = 0.927 respectively. Competence scale with 6 items was reliable at α = 0.768. The prosocial subscale consisted of 3 items (α = 0.821) and antisocial sub-scale consisted of 5 items (α = 0.719). Internal validity was met by eliminating confounding variables.

### 2.3. Procedures

The university institutional review board approved all study procedures. A quasi-experiential design with an initial questionnaire, intervention activity, and final questionnaire was administered to students at a summer camp. The intervention activity was throwing and catching a football. A pre-test was done at the beginning of the two-week period of intervention. A post-test was administered at the end of two weeks. The questionnaires were distributed in the gym where the campers were doing their intervention activities. Administering the surveys took an average of 15 min. Those campers who could not comprehend some items were helped to interpret the meanings.

### 2.4. Data Analysis 

Data analysis was conducted using descriptive statistics, correlation, and regression and mediation in SPSS 24.0 [22]. Correlation analysis was done to assess the association between situational interest, personal interest, and competence in a throwing and catching a football. Regression was then conducted to assess the mediation role of prosocial behavior in the relationship between situational interest and personal interest. A Sobel test was then run to test for the significance mediation effect of social support on the relationship between personal interest and situational interest. A paired-sample *t*-test was conducted to compare personal interest (FPI) and situational interest (FSI) in football (Table 1). A personal interest questionnaire was administered before the activity commenced, while the situational interest questionnaire was administered after the intervention activity.

## 3. Results

Prior to running multiple linear regression analysis, correlation analysis was done to ascertain the relationship between personal interest, situational interest, and competence. Independent sample T-tests were run to assess difference between boys’ and girls’ level of situational and personal interest. Independent sample T-Test results show insignificant difference in boys’ (M = 3.891, *SD* = 0.934) and girls’ (M = 3.995, *SD* = 0.893) level of situational interest; t (38) = −0.222, p = 0.827. In addition, the independent sample T-test found significant difference between boys’ (M = 3.529, *SD* = 1.296) and girls’ (M = 2.147, *SD* = 1.225) personal interest; t (38) = –3.409, p = 0.002. 

Results point to a correlation between the three variables in Table 1. Specifically, our results suggest that the three variables are positively related, even though we cannot tell how each one affects the other. 

The results of the multiple linear regression in Table 2 indicated that there was a collective significant effect between the prosocial behaviors, perceived competence, personal interest, and situational interest (F(3, 39) = 41.29, *p* < 0.001, *r*^2^ = 0.76). Further examination of individual predictors indicated that perceived competence (*t* = 7.07, *p* = 0.001) and personal interest (*t* = 3.05, *p* = 0.004) were significant predictors in the model. An analysis of prosocial behavior revealed a barely significant relationship (*t* = 2.02, *p* = 0.051), suggesting that this variable might be the mediator in the relationship between personal interest and situational interest. 

Two regression tests were conducted to determine the mediation effect of prosocial behavior on personal interest and situational interest. In Step 1 (Table 3) of the mediation model, the regression of personal interest on situational interest, ignoring the mediator, was significant (*b* = 0.674, *t* (39) = 5.492, *p* = 0.001). 

In Step 2 (Table 4) of the model with two predictor variables (personal interest and prosocial behavior), it was shown that the regression of prosocial behavior on situational interest was insignificant (*b* = 0.194, *t* (39) = 1.178, *p* = 0.246). Whenever there is a real mediator effect, the predictor variable should not be significant in the model, when the mediator is included. From these results, the predictor variable is significant, suggesting that prosocial behavior is not a mediator in the relationship between personal interest and situational interest. The Sobel test [23] results were insignificant (*p* = 0.249).

## 4. Discussion and Conclusions

This study investigated the complex relationship between personal interest, situational interest, competence, and prosocial patterns among children enrolled in summer camp. Researchers have often argued that there is a direct relationship between situational interest and personal interest. This relationship suggests that their interest is a major component of motivation [9]. Studies examined show that situational interest is affected by social factors [24]. More specifically researchers [11] linked situational interest to basic psychological needs of competence, autonomy, and relatedness.

Participants in this study reported a higher situational interest than personal interest. The implication of this outcome points to the role of social factors in the camping environment. It can be suggested that the social environment, specifically the peers and camp counselors, influenced the campers’ situational interest. The social environment supported autonomy, relatedness, and competence. The number of times campers had attended this camp affected the results, as some of them already had previous camping experience upon which they built their situational interest. In addition, the strong correlation between personal interest and situational interest supports previous findings which have shown that the two variables stem from one factor and are closely linked [8,9].

Whereas situational interest is a temporary interest that occurs spontaneously due to environmental factors such as task instructions, personal interest is a relatively permanent preferences usually expressed in different contexts [7]. Independent sample T-test results suggest that even though boys had higher personal interest in the throwing and catching task, there was no significant difference between boys’ and girls’ situational interest. Since situational interest is dependent on task instruction, it seems camp counsellors’ pedagogical approaches provided learning environment that triggered and maintained situational interest for all the participants irrespective of gender. It is also possible that the camping environment eliminated the effect of personal interest to the campers. These findings are important because they eliminate any concerns that even though there was higher personal interest in throwing and catching task amongst boys than girls, there was no significant difference in situational interest. 

Pearson correlation coefficient and multiple linear regression results point to the relationship between competence and situational interest. These results supported a theoretical approach which hypothesized that competence positively influences situational interest [9]. Campers who consider themselves competent in the tasks provided at the camps are more likely to be interested and motivated to stay engaged in the activity. In this sample, it seems that, at the end of the intervention period (three weeks), most of the campers perceived themselves as competent. They were able to engage in the activity, but they were also motivated to do so.

The results further suggest that personal interest, competence, and prosocial behavior are predictors of situational interest. The strong correlation between the prosocial behavioral pattern and situational interest suggest that when camper counselors establish conditions that support situational interest, this is likely to influence prosocial behaviors. A social environment that is supportive of elementary-age summer campers’ results in an increase in traits of respect, good communications, and positive interactions with others.

Contrary to the hypothesis, it was found that prosocial behavior does not mediate the relationship between personal interest and situational interest. This is inconsistent with literature (5, 6) that has suggested that this relationship might be mediated by prosocial behavior. Therefore, prosocial behavior has a positive effect on situational interest, but does not mediate the relationship between personal interest and situational interest.

## 5. Implications

This study has implications for parents, camper organizers, camp counselors, the tourism industry, and researchers. Parents that allow children to attend summer camps help them develop efficient physical and social skills. It is important for camp counselors to establish a camping environment and activities that trigger and maintain participants’ interest. This can be achieved by encouraging interpersonal relations among students (relatedness) and helping all the campers acquire the competences they need to participate in the assigned activities. The tourism industry will need to continue to invest in the level of interest in camping activities. For researchers, this study has provided insight into the relationship between interest, the social environment, competence, and behavior.

This study had several limitations. First, the study population was children enrolled in a summer camp and hence the results might not be expanded to the entire population. Secondly, this study focused on only one task (football), which might be biased towards some participants [25]. Lastly, this study was conducted in a controlled learning environment (gym) and this might have had an impact on the way participants behaved.

One of the limitations of this study was finding literature related to interest theory and prosocial behavior in settings outside physical education. Most of the past research was done in physical education context. However, from this study it can be seen that interest theory and pro-social support are valuable tools applicable in summer camps. 

## 6. Recommendations

The power of competence and situational interest of elementary-age campers can be beneficial for parents to choose the best summer camp that fits their child. This study supports summer camp owners and counselors establishing a curriculum that encourages pro-social skills based on personal and situational interest. One recommendation would support a theoretical approach to the development of summer camp curriculum that supports the key notion of pro-social skills and physical skills competencies based on each camper’s interest. Past research has already pointed to the importance of structured out-of-school time [4], but the present research supports a strong foundation in developing competency in physical and social skills [26]. Despite the interest of the public and present research on the relationship between interest and competencies, the established curriculum has not examined variations in what activities children who attend summer camps are most competent in. 

The development of a positive framework for the summer camp curriculum should first define competencies in physical and pro-social skills. This study contributes to the examination of teaching and learning practices that contribute to the development of the key competencies in physical and pro-social skills. Recent research [15] supports the argument that students’ perception of their performance can be enhanced by the interpersonal skills of teachers.

Further studies could focus on how teachers in summer camps contribute to the development of elementary-age summer campers’ competence in physical and pro-social skills. Finally, the results convey that identifying the specific situational interest is important when designing a prosocial environment to help elementary summer campers acquire new competences that they can transfer to society.

## Figures and Tables

**Table 1:** Correlation among Situation Interest, Personal Interest, and Competence.

		PI	SI	Com
PI	Pearson Correlation	1		
	Sig. (2-tailed)			
	N	40		
SI	Pearson Correlation	0.665 **	1	
	Sig. (2-tailed)	0.000		
	N	40	40	
Com	Pearson Correlation	0.565 **	0.833 **	1
	Sig. (2-tailed)	0.000	0.000	
	N	40	40	40

** Correlation is significant at the 0.01 level (two-tailed). Note: PI = personal interest; SI = situational Interest; and Com = competence.

**Table 2:** Multiple regression results of personal interest, situational interest, and prosocial behavior.

Model		Unstandardized Coefficients		Standardized Coefficients	*t*	Sig.
		B	Std. Error	Beta		
1	(Constant)	−0.143	0.497		−0.288	0.775
	mean FPC	0.676	0.096	0.678	7.065	0.000
	mean FPI	0.297	0.097	0.293	3.052	0.004
	mean PS	0.219	0.108	0.160	2.023	0.051

Note: FSI = situational interest; FPC = perceived competence; and PS = prosocial behaviors.

**Table 3:** Regression of personal interest on situational interest.

Model		Unstandardized Coefficients		Standardized Coefficients	t	Sig.
		B	Std. Error	Beta		
1	(Constant)	1.816	0.401		4.534	0.000
	mean FPI	0.674	0.123	0.665	5.492	0.000

Dependent variable: FSI (situational interest). Predictor variable: FPI (personal interest).

**Table 4:** Regression of personal interest and prosocial behavior on situational interest.

Model		Unstandardized Coefficients		Standardized Coefficients	T	Sig.
		B	Std. Error	Beta		
2	(Constant)	1.131	0.706		1.603	0.117
	mean FPI	0.684	0.122	0.675	5.586	0.000
	mean PS	0.194	0.165	0.142	1.178	0.246

Dependent variable: mean FSI (social support). Predictor variables: FPI (personal interest) and PS (prosocial behavior).
